# Nanofire and scale effects of heat

**DOI:** 10.1186/s40580-019-0175-4

**Published:** 2019-02-15

**Authors:** Zhimao Wu, Gang Yang, Erzhen Mu, Qiuchen Wang, Sebastiaan A. Meijer, Zhiyu Hu

**Affiliations:** 1 National Key Laboratory of Science and Technology on Micro/Nano Fabrication, Shanghai Jiao Tong University, Shanghai, 200240 China; 2 Institute of NanoMicroEnergy, Shanghai Jiao Tong University, Shanghai, 200240 China; 3 Department of Micro/Nano Electronics, Shanghai Jiao Tong University, Shanghai, 200240 China; 4 KTH Royal Institute of Technology, Stockholm, Sweden

**Keywords:** Nanofire, Thermal gradient, Scale effects of heat

## Abstract

Combustion is a chemical reaction that emits heat and light. Nanofire is a kind of flameless combustion that occurs on the micro–nano scale. Pt/Al_2_O_3_ film with a thickness of 20 nm can be prepared as a catalyst by micro–nano processing. When the methanol-air mixture gas passes through the surface of the catalyst, a chemical reaction begins and a significant temperature rise occurs in the catalyst region. Compared to macroscopic combustion, Nanofire has many special properties, such as large temperature gradients, uniform temperature distribution, and fast temperature response. The large temperature gradient is the most important property of Nanofire, which can reach 1330 K/mm. Combined with thermoelectric materials, it can realize the efficient conversion of chemical energy to electric energy. Nanoscale thickness offers the possibility of establishing thermal gradient. On the other hand, large thermal gradient has an effect on the transport properties of phonons and electrons in film materials. From these we can get the scale effects of heat. This article will provide an overview of the preparation, properties and applications of Nanofire, and then a comprehensive introduction to the thermal scale and thermal scale effects.

**Electronic supplementary material:**

The online version of this article (10.1186/s40580-019-0175-4) contains supplementary material, which is available to authorized users.

## Introduction

Fire is one of the earliest energy sources used by human beings [1]. It has been affecting the evolutionary steps of human beings from the very beginning. It can be said that the history of human development is the history of human beings using fire and transforming fire [2, 3]. Until now, a large part of the energy used by humans still comes from combustion [4]. The fires we use today are mostly on macro scale and in three dimensions. In fact, the fire is difficult to produce in small scale (< 1 μm). At the same time, with the development of society, environmental pollution and energy depletion have gradually become two major problems in the world. How to use energy clean and environmental friendly has become a social problem that needs to be solved urgently [5]. Catalytic combustion is more stable than traditional combustion, and it has fewer pollutant emissions [6]. What’s more, we can greatly improve the utilization rate of combustible gas with the catalytic combustion. Because of that, catalyst combustion has attracted the attention of the academic community and the whole society. Conventionally, we prepare catalysts by chemical methods such as immersion pulling, precipitation deposition and sol–gel [7–9], Bonet et al. have synthesized Au, Pt, Pd, Ru and Ir nanoparticles with a narrow size distribution by chemical reduction of their corresponding metal species in ethylene glycol. But it is difficult to control the position, size and morphology of the catalyst obtained by these methods and the catalyst is easily deactivated at a high temperature [10], Asoro et al. used STEM to study the agglomeration and deactivation of catalyst during the reaction, then they got the catalyst to be easily aggregated and deactivated at high temperatures. By means of micro–nano processing technology, Somorjai et al. proposed that we can prepare catalyst films with a thickness of nanometers and we can control their size, shape and position [11–13]. Previously, we have prepared different types of nanofilm catalysts by ultraviolet lithography and magnetron sputtering [14] or inkjet printing [15, 16], and the combustion of methanol-air mixture gas can occur at room temperature with these catalysts [6, 17]. This kind of combustion has many characteristics different from macro-scale combustions [14], we call it as Nanofire. It can be found through experiments that Nanofire has a very uniform temperature distribution and a rapid temperature change response to the flow rate of methanol gas. At the same time, it was found that the catalyst region had a temperature rise of about 12 °C so that an extremely large temperature gradient could be realized on the film of 20 nm thick. In order to further improve the catalytic performance of the catalyst, we also prepared particle catalyst with a diameter of 50 nm and a height of 30 nm by electron beam lithography. It was found by experiments that the particle catalyst has better catalytic properties than film catalyst. Large temperature gradients combined with thermoelectric materials can convert chemical energy into electrical energy [18, 19], Dechaumphai et al. have studied that the ultra-low thermal conductivity of multilayer thin film materials can improve the thermoelectric properties of thermoelectric materials. Other studies have also shown that thin film thermoelectric materials have better thermoelectric properties than bulk material [20–22]. Meanwhile the development of micro–nano technology makes the integration of Nanofired power generation devices possible [23].

When the characteristic length of the material is smaller than the mean free path of the carrier, including electrons and phonons, the continuous medium assumes failure, and the existence of the materials interface makes the Fourier heat conduction law no longer applicable [24–26]. Scale effects in thermal transfer originated from the experimental work of Haas and Biermasz et al. corresponding theoretical explanation had been given by Casimir [27, 28]. Subsequently, Ziman analyzed the size effects based on Boltzmann transport equation [29, 30]. After that, Tien et al. promoted and developed micro–nanoscale heat transfer research from experimental measurement to model analysis systematically [31–33]. Up to now, there are a lot of groups devoted to micro–nanoscale heat transfer studies. The newest micro–nano-scale heat conduction theory has also proved that the thermal conductivity decreases rapidly as the material size decreases in the nano scale [34–37], McGaughey et al. presents a formulation for studying the thermal transport in dielectric materials using MD simulations, and then the relationship between thermal conductivity and materials size is obtained. Whlie Peierls et al. proposed the relationship between thermal conductivity change and solid size by studying the lattice of solid materials. For the film materials appearing in this manuscript, the mean free path of both phonons and electrons is much larger than the thickness of the material, so we need to consider the scale effect of heat transfer. The scale effects cause the appearance of thermal gradients, and the large thermal gradient also affects the transport of carriers such as phonons and electrons in materials, we collectively refer to the scale effects of heat. In the next part of this manuscript, the preparation methods, characteristics and applications of Nanofire will be introduced. Then the thermal effects of Nanofire will be introduced, from that we can get the scale effects of heat transfer. We want to take Nanofire as an example to explore the scale effects of heat and heat transfer. Finally, a potential method of exploring the future effects of Nanofire is introduced.

## Preparation of Nanofire

In order to realize the controllable characteristics of Nanofire, this manuscript will focus on three preparation methods. Firstly, the catalyst can be directly deposited by inkjet printing technology. Secondly, we can use ultraviolet lithography for pattern transfer and then magnetron sputtering for film deposition. Lastly, we can get the catalyst by electron beam lithography.

**Fig. 1:**
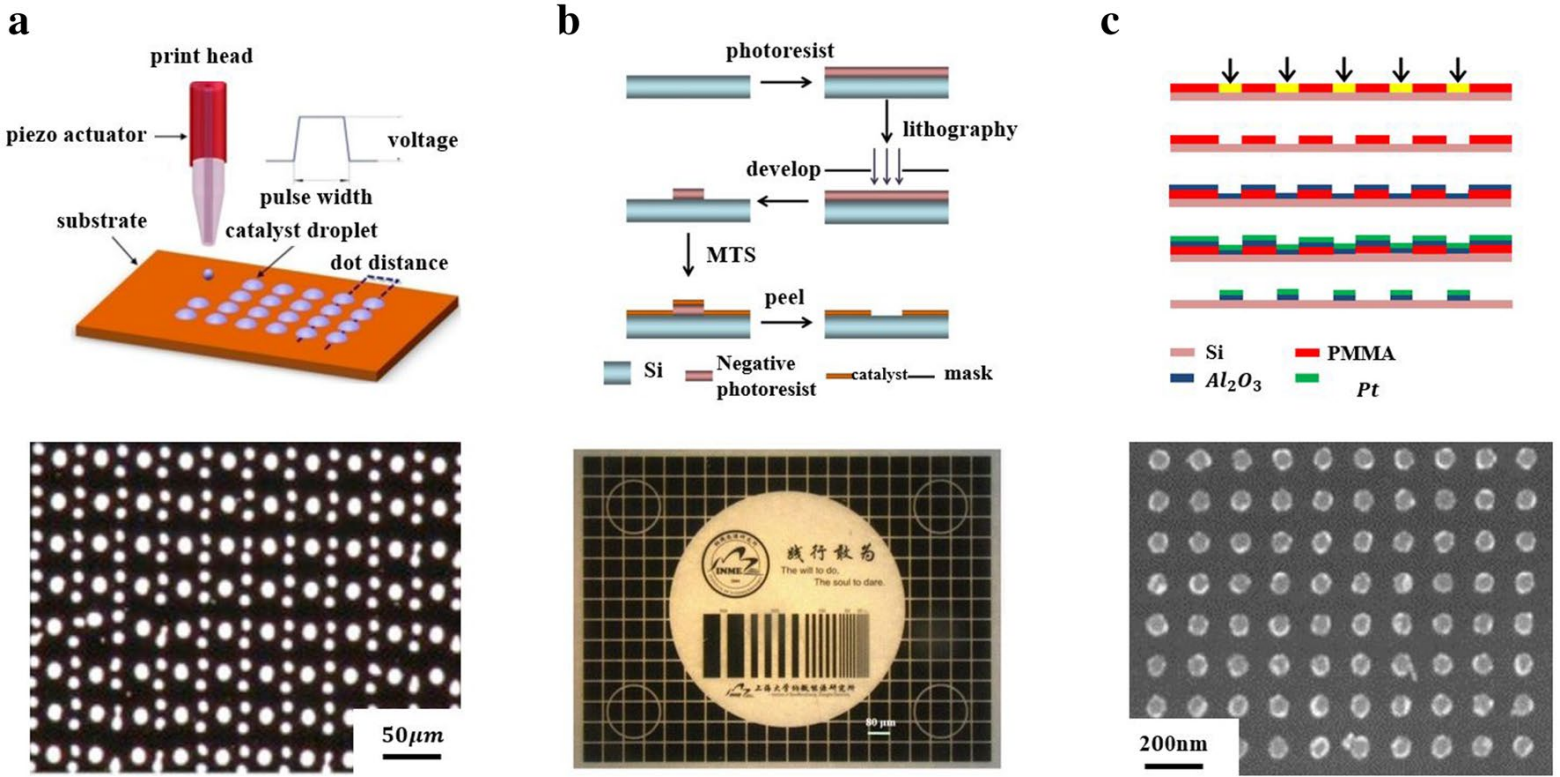
Preparation of catalyst by micro–nano processing. **a** Inkjet printing, **b** film deposition, **c** electron beam lithography, **d** SEM of catalyst by inkjet printing, **e** optical image of catalyst by film deposition, **f** SEM of catalyst by EBL

Inkjet printing is a technique for microscale patterning and film deposition on specific substrate materials [15, 16]. In the process of preparing a catalyst by inkjet printing, the platinum catalyst was synthesized in situ on a substrate. An ink jet printable solution containing platinum ions was prepared by dissolving a commercially available chloroplatinic acid powder in water. The printing ink has a concentration of 0.01 mol/L. The catalyst precursor solution is then printed on a heated substrate with a particular shape and defined loading. The inkjet printer used as a micropatterning tool in this experiment was a commercial Autodrop microdispensing system (Microdrop Technologies GmbH, Germany). Applying voltage and pulse width are two key factors in determining the printability and size of a droplet, it was previously used to prepare catalyst films by optimizing inkjet-printed parameters (voltage: 65–70 V, pulse width: 20–30 μs, ink viscosity: near 1 mPa s) and depositing uniform morphologies (substrate temperature: 80 °C; water/ethylene glycol (W/EG): 95:5, v/v). As shown in Fig. 1a, d, no mask is required compared to conventional lithography, resulting in lower equipment and time costs. It has broad development prospects and has many applications in field effect transistors, light emitting diodes, fuel cells, and various micro/nano scale sensors.

We can more accurately control the shape, position and size by ultraviolet lithography combined with magnetron sputtering technology for graphical film deposition [14]. As shown in Fig. 1b, e, a specific thickness of the photoresist is applied on the silicon wafer, then the pattern is transferred by photolithography. After that, patterned catalyst film is obtained by subsequent coating and stripping processes, the surface morphology of the catalyst can be analyzed by SEM and AFM, and the longitudinal structure and composition of the catalyst film can be verified by TEM. Prior to photolithography, the oxide layer on the surface of the silicon substrate (100) was removed using HF and washed with deionized water. During the sputter coating process, the pressure of the sputtering chamber is maintained at 10^−6^ Torr, and the surface roughness of the film can be controlled by adjusting the ratio of oxygen to argon. In this experiment, the ratio of oxygen to argon was set to 2%, and the sputtering power was set to 100 W. The thickness of the sputtered aluminum oxide film was 20 nm, the thickness of the platinum catalyst film was 5 nm, and the film thickness was controlled by controlling the sputtering time.

We can process and modify the morphology of the catalyst at a size of 10 nm by electron beam lithography. The particle catalysts described in this manuscript are mainly platinum nanoparticles with a diameter of 50 nm, which has a larger effective catalytic area and better catalytic activity than film catalysts. Electron beam lithography is similar to UV lithography, but it does not require a mask. As shown in Fig. 1c, f, a 120 nm thick standard poly methyl methacrylate (PMMA) photoresist was used. During the lithography process, the pattern was written into the PMMA layer by a high collimated electron beam (Leica Nano writer) generated by a field emission source. The dose is 600 μC/cm^2^. Then the PMMA photoresist written by the electron beam undergoes a denaturation reaction to complete the transfer of the pattern.

Advances in micro–nano processing technology have allowed us to design devices and related structures at micro–nano scales, various micro-scale sensors are playing a role in more and more applications. As micro–nano processing technology can be used at smaller scales, the sensors made by these methods have a greater advantage, as for our Nanofire.

## Characteristics of Nanofire

**Fig. 2:**
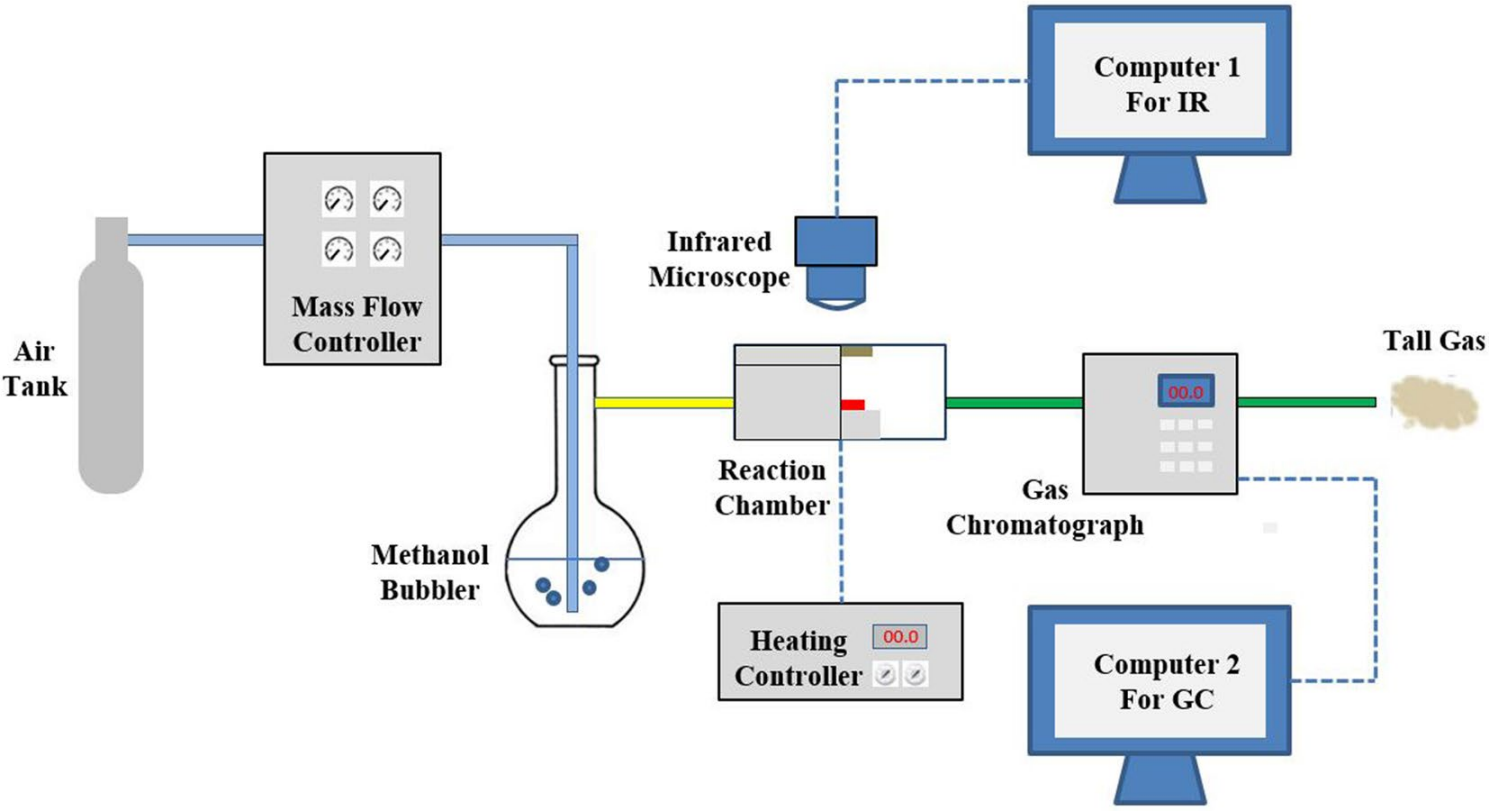
The detection device of catalytic combustion

Compared with traditional combustion, Nanofire has many special properties. Firstly, it can burn at room temperature. In addition, no flame is generated in the combustion process. Due to the special law of micro–nano-scale heat transfer, the heat generated by the combustion reaction can accumulate rapidly on the surface of the catalyst, resulting in an extremely large thermal gradient. Otherwise, the shape and size of the catalyst are controllable. Therefore, the temperature distribution of the Nanofire is relatively uniform. Because of the existence of the scale effect, the temperature change is sensitive to the combustion, so the catalytic performance can be characterized by the temperature change of the catalyst surface. As shown in Fig. 2, we can get the catalytic performance with these detection devices. The flow rate of methanol can be controlled by the gas mixing device, the change of the surface temperature can be visually observed by the infrared microscope, and we can quantitatively calculate the conversion efficiency of the methanol by the gas chromatograph. The surface morphology, temperature distribution, temperature response, and temperature gradient of the catalyst are described in the next parts of this section.

### Characterization of catalyst

**Fig. 3:**
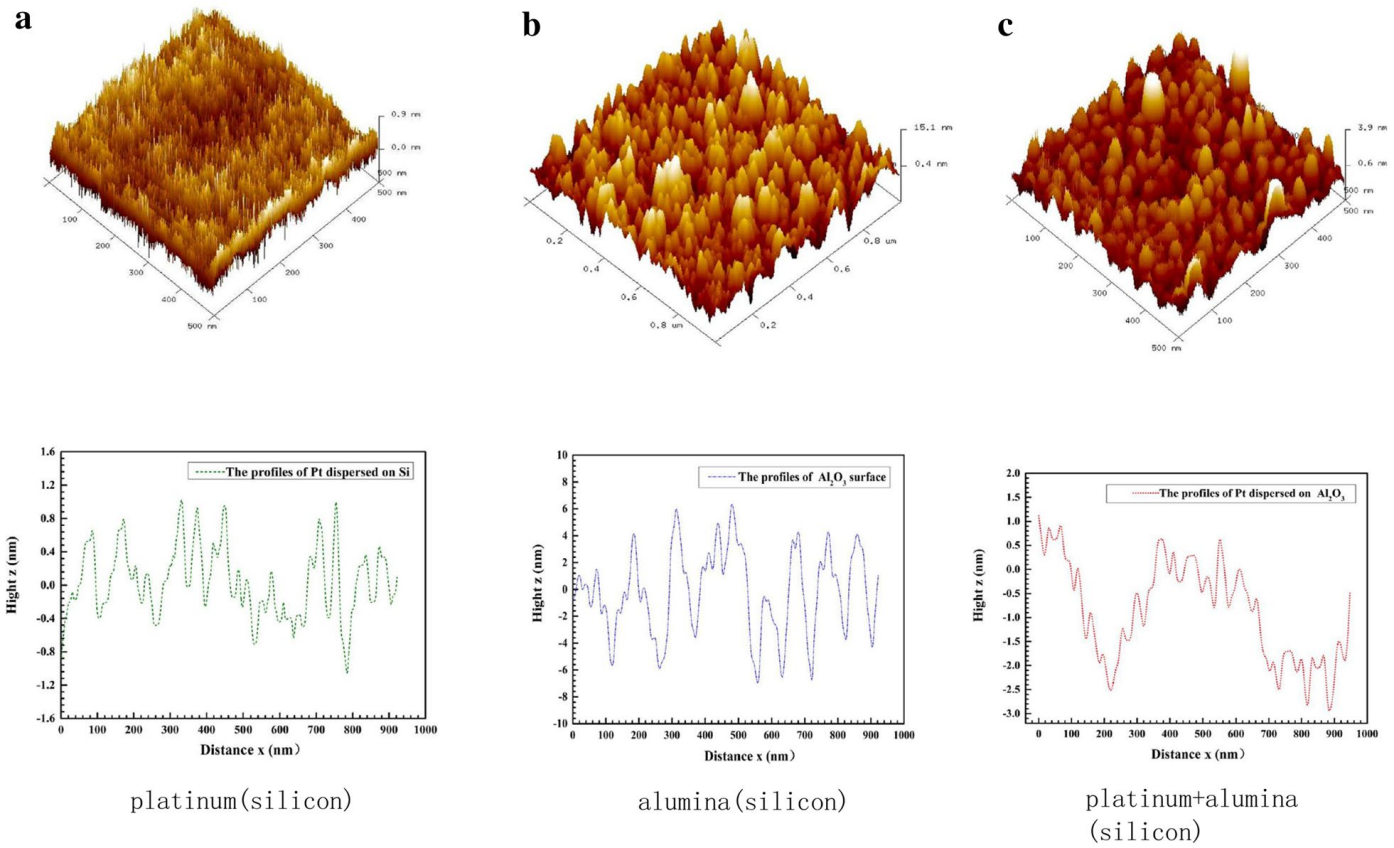
AFM image of film catalyst (from left to right, platinum deposited on a silicon substrate, alumina deposited on a silicon substrate, and platinum deposited on alumina)

The surface morphology of the film catalyst was characterized by atomic force microscope, then the surface roughness of the catalyst was obtained. During the experiment, different regions were repeatedly scanned to obtain a more accurate morphology. For comparison, three sets of samples were selected, platinum deposited directly on the silicon substrate, alumina deposited on the silicon substrate, and platinum deposited on the alumina. As shown in Fig. 3, we can see that the thickness of alumina and platinum is around 20 nm. And platinum deposited on a silicon substrate is with a very low roughness, while alumina deposited on a silicon substrate with a relatively high roughness value. Under the same conditions, platinum deposited on the surface of alumina shows moderate roughness, mainly because the surface roughness of alumina forms a valley region that can accommodate platinum particles, which can also effectively reduce the agglomeration of platinum particles.

**Fig. 4:**
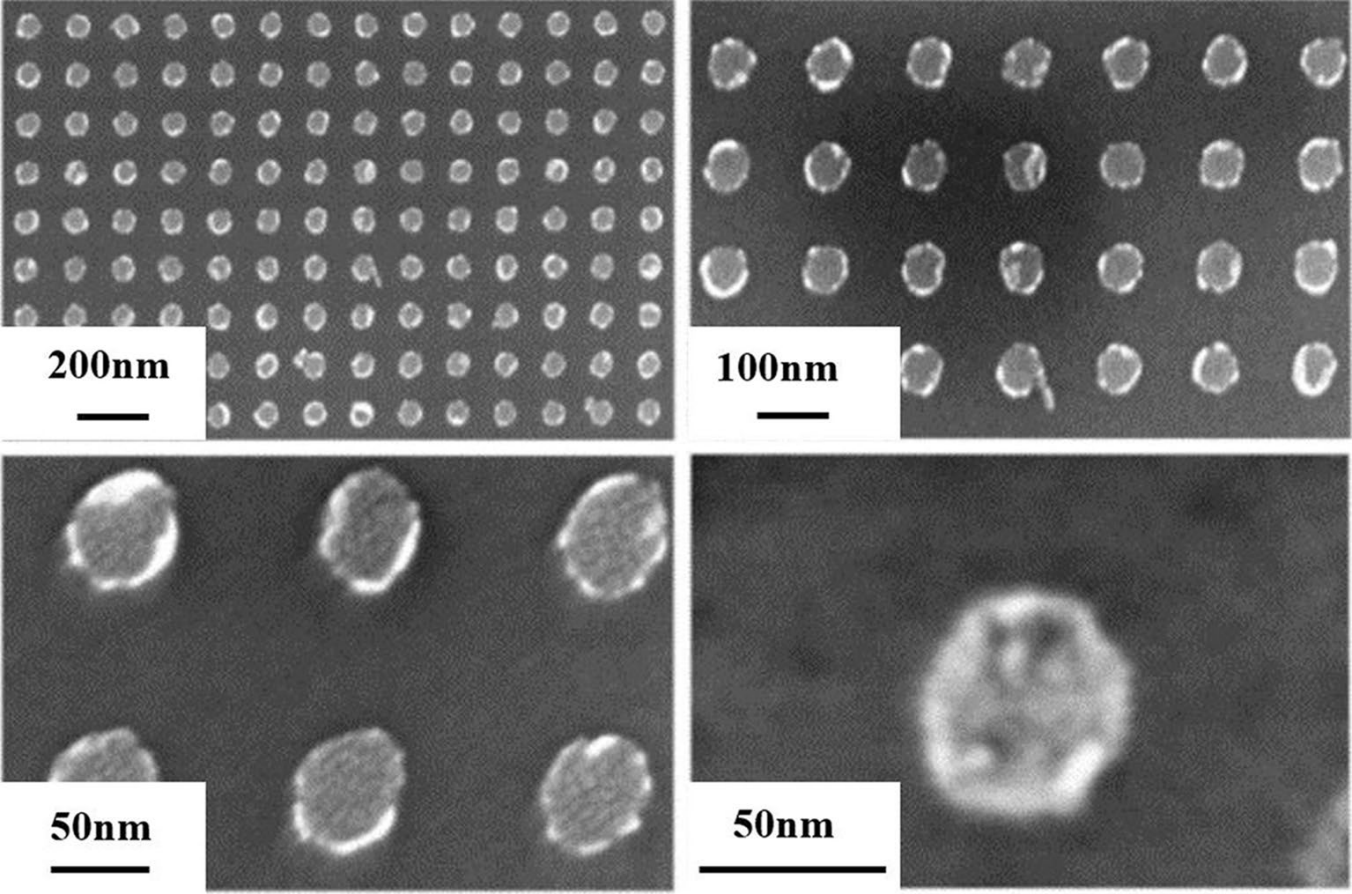
SEM image of particle catalyst (different scales)

The surface morphology of the particle catalyst can be characterized by SEM. In this manuscript, the particle catalyst has a diameter of 50 nm. As shown in Fig. 4, the particle diameter is 50 ± 2 nm, the center-to-center distance is 150 ± 3 nm. It shows that the sample is successfully prepared.

### Catalyst performance and influencing factors

**Fig. 5:**
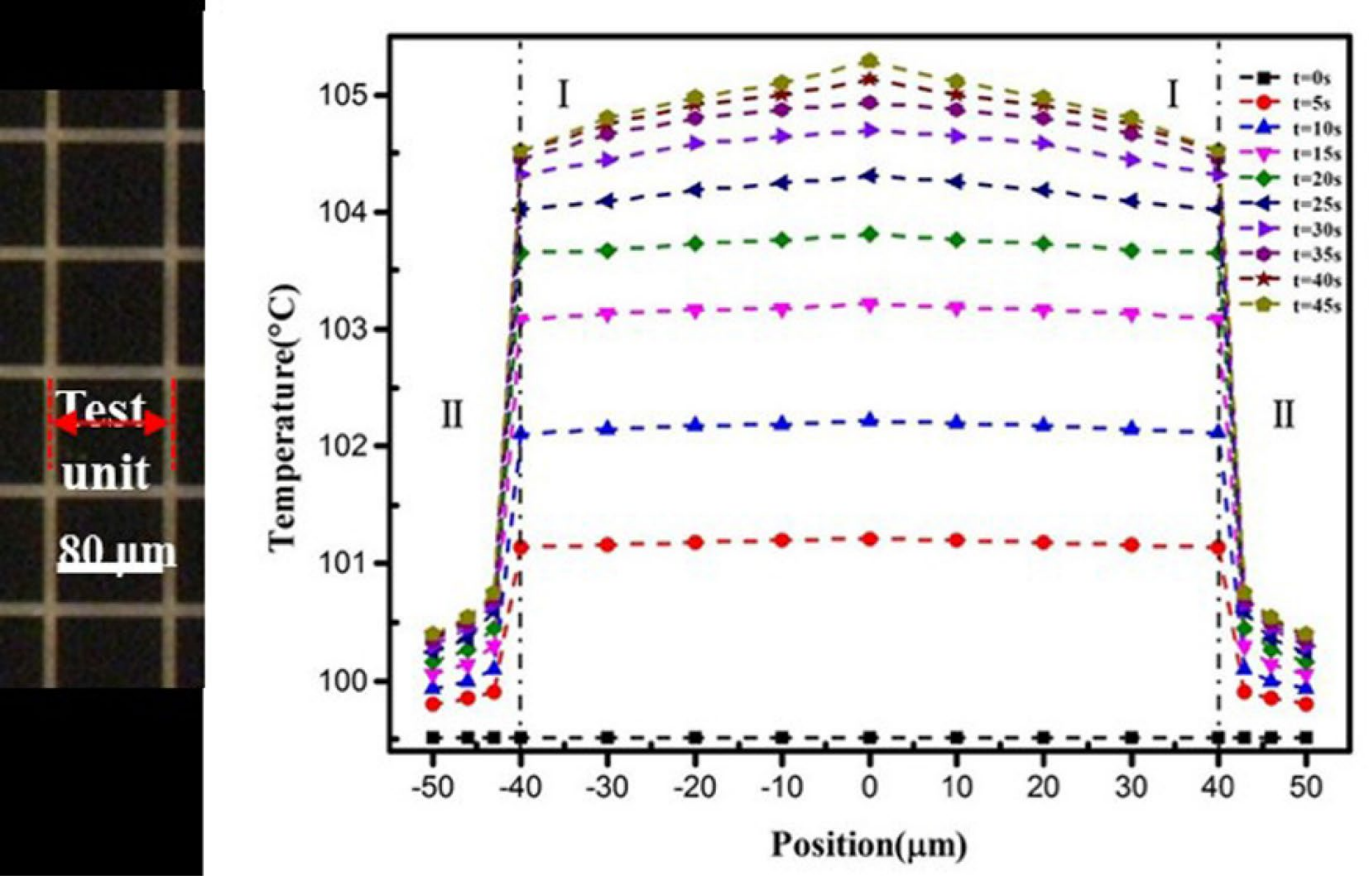
The surface temperature distribution of platinum catalyst

In order to study the temperature distribution of the Nanofire, an infrared microscopy is used, it has sub-micron spatial accuracy and a temperature accuracy of 1 K. The observation area was 800 μm × 800 μm. The results obtained are shown in Fig. 5. As the reaction progresses, the temperature of the catalyst region rises rapidly and tends to be stable. It can be clearly seen that the temperature distribution of the entire catalyst region is uniform and consistent, which indicates that the Nanofire can be used as a stable micro heat source to provide stable heat output. Particle catalysts have similar properties but higher catalytic properties.

**Fig. 6:**
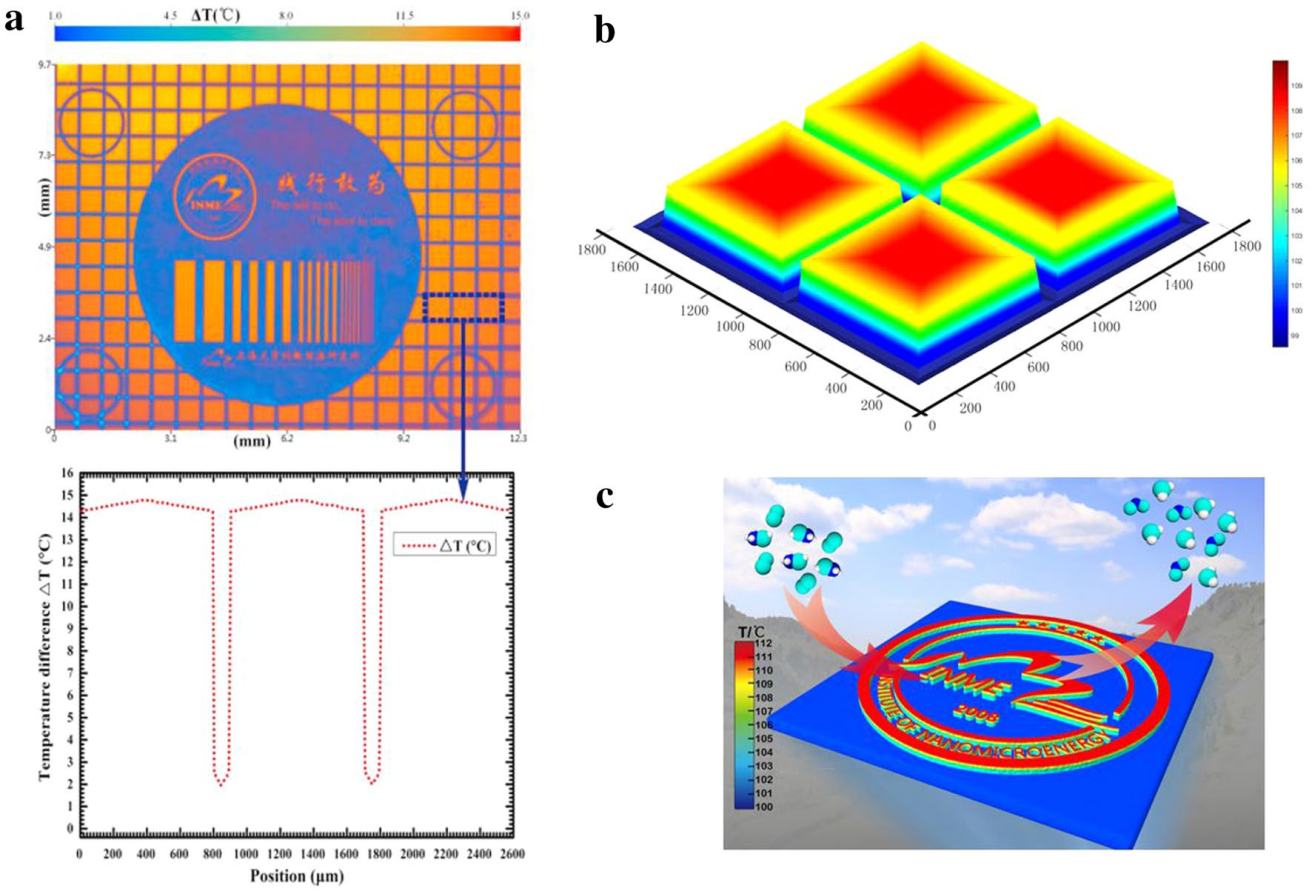
Two-dimensional Nanofire. **a** Infrared microscope image, 12 mm × 10 mm, different colors indicate different temperatures; **b** simulation diagram of Nanofire; **c** diagram of catalytic reaction and Nanofire

As the chemical reaction proceeds, the generated heat rapidly accumulates in the catalyst region, causing a large temperature increase. Meanwhile the temperature of the none catalyst region is only slightly increased, so a relatively large thermal gradient is formed in the vertical direction of the substrate, and Fig. 6 can be obtained by infrared microscopy. The results show that a temperature difference of more than 10 K occurs at a thickness of 20 nm, and the temperature difference can be stably present, which is impossible to achieve on a macroscopic scale.

In addition, the large temperature gradient formed by the Nanofire makes it possible to draw a fire pattern at the nano scale. As shown in Fig. 6, we can control the pattern indirectly by controlling the position, shape and size of the catalyst. We have prepared patterns such as “INME” by micro–nano processing technology before, when methanol passes, the combustion reaction occurs on the surface of the catalyst, the heat is rapidly accumulated, and a large temperature gradient is established. Then the fire is graphical.

## The application of Nanofire

**Fig. 7:**
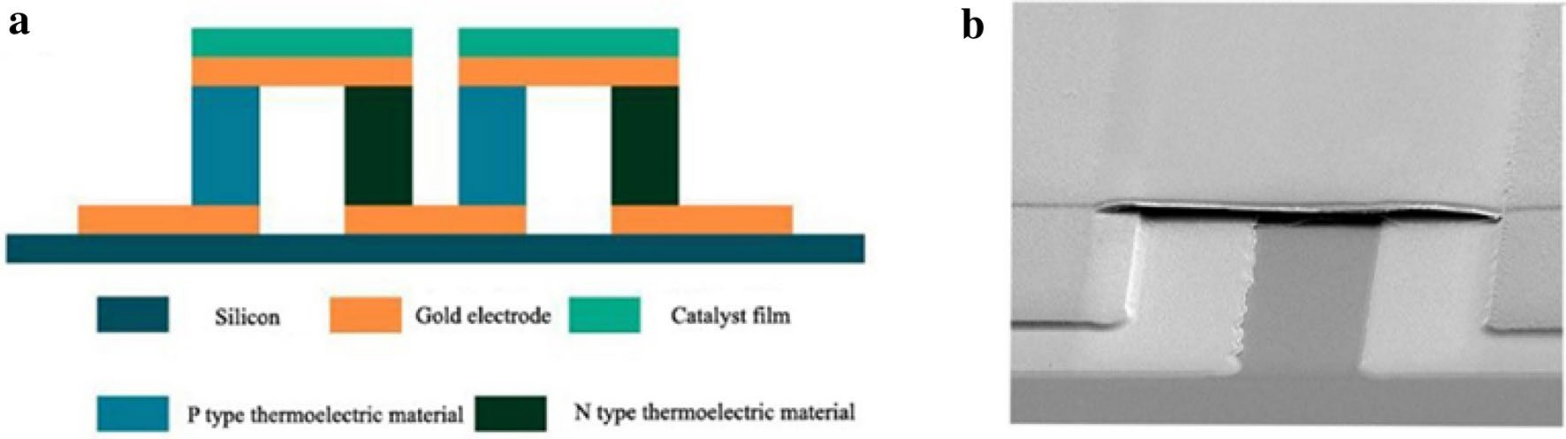
**a** Schematic diagram of the thermoelectric chip, **b** SEM of thermoelectric chip

In order to achieve high-efficiency application of Nanofire, a certain number of thermoelectric pairs are prepared on the substrate, then the catalyst is deposited on the thermoelectric pair by photolithography and film deposition. As shown in Fig. 7, the thickness of the electrode and catalyst is of nanometers, and the size of the thermoelectric material is of micrometers. Then, large-scale integration forms a thermoelectric chip. As shown in the previous experimental results, since there is a scale effect of heat transfer at the nanometer scale, we can simplify the power generation part into a four-layer structure, catalyst-electrode-thermoelectric film-electrode, and contact thermal resistance will appear on any two layers. Therefore, there will be a large temperature difference between the hot and cold ends of the thermoelectric material. This will improve the thermoelectric conversion efficiency of thermoelectric materials.

When the gas mixed with methanol air flows through the surface of the chip, the whole chip becomes a micro power plant, and only a small amount of methanol is needed to generate a large amount of electricity. We can make a simple calculation, assuming that 40,000 pairs of thermoelectric columns can be prepared on a four-inch silicon wafer. The seebeck coefficient of the thermoelectric material is 300 μV/K. Assuming that the temperature difference between the thermoelectric material is 1 K, then each thermoelectric pair can get a voltage of 300 μV, and the entire thermoelectric chip can get a voltage of 12 V. Through our calculations, achieving a 1 K temperature gradient requires little methanol, about 1 mL/min. And we also proved this through experiments.

**Fig. 8:**
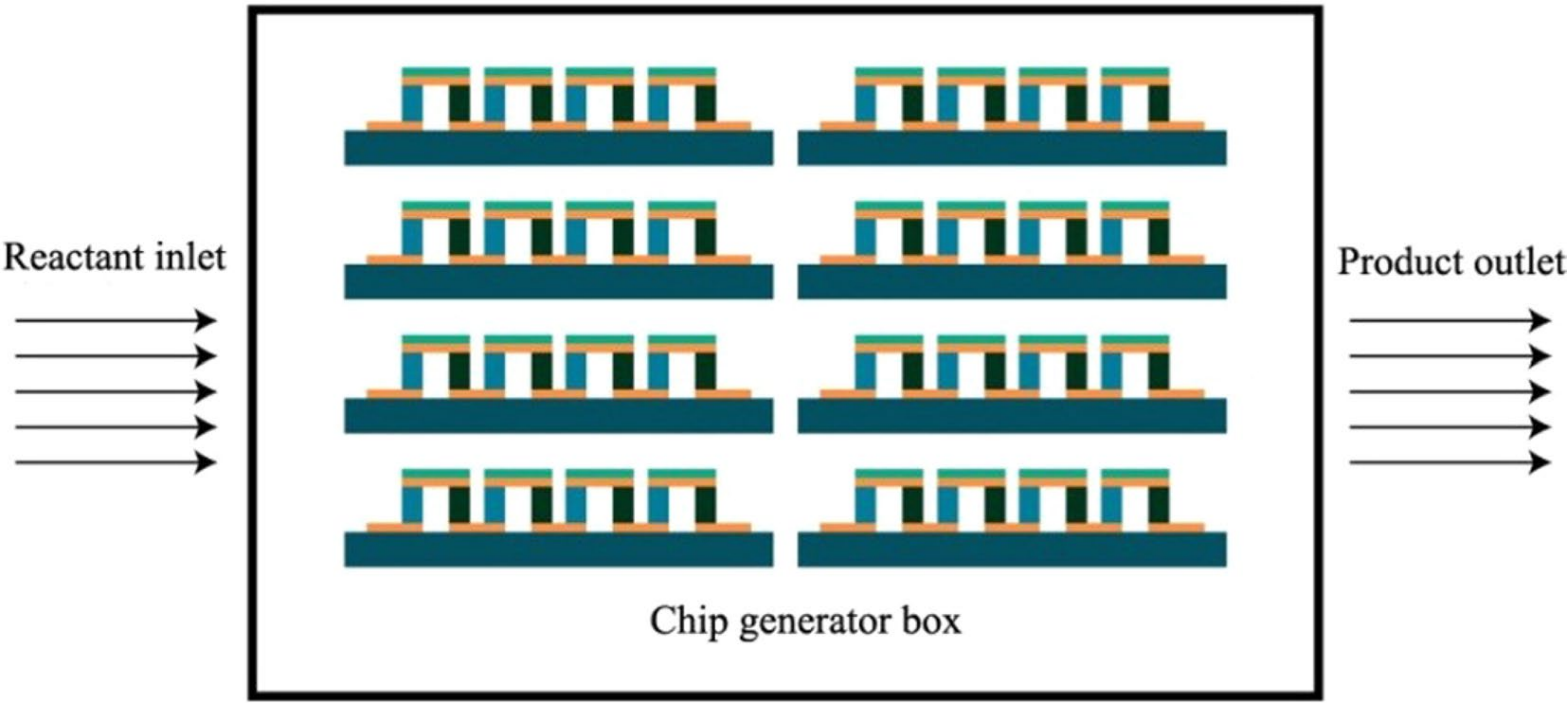
Schematic diagram of an integrated packaged chip generator box

As the technology matures, thermoelectric chips can be made larger, and tens of thousands of thermoelectric chips can be connected in series, so that a chip power generation plant can be formed. The combustion reaction product of the methanol is only carbon dioxide and water, and there is no danger of explosion and leakage during the reaction process. In addition, the catalytic combustion reaction is relatively stable, so that the chip can have a stable power generation capacity. Studies have also shown that the thermoelectric properties of multilayer film thermoelectric materials have been greatly improved at the micro–nano scale, which further improves the conversion efficiency of thermal energy to electrical energy. As shown in Fig. 8, the high-integration package of the multilayer thermoelectric chip can get a large current output when the methanol-air mixture passes. In the future, thermoelectric chips based on Nanofire and thermoelectric materials will be able to support the daily use of electricity in a home, and even provide electricity for industrial production.

## Thermal gradient formation

**Fig. 9:**
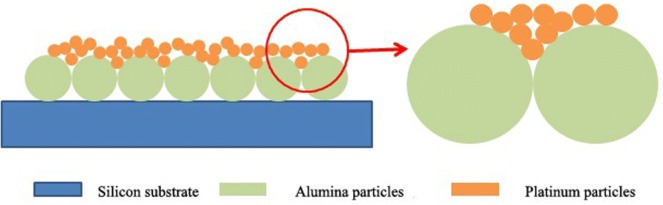
Schematic diagram of contact between alumina particles and platinum particles

One of the most striking features of Nanofire is the large temperature gradient, which is difficult to achieve at the macro scale. Because of the scale effect, the heat generated by chemical reaction can accumulate rapidly on the upper surface of the catalyst, and its downward conduction is very small. Regarding the heat conduction law of micro–nano scale, many scholars at home and abroad have proposed their own calculation methods, including equilibrium or nonequilibrium states molecular dynamics simulation based on micro-scale calculation [34] and first-principles simulation based on density-functional theory calculations with plane waves and pseudopotential [35]. The theory of sub-motion theory [36] and the phonon hydrodynamic model derived from the kinetic theory of phonons for nanoscale heat transport [37], the two-phase lag model [38], the ballistic-diffusion model which approximate analytical solution of Boltzmann transport equation [39] and the hot gas model are more mainstream [40]. For Nanofire, since the thickness of the aluminum film and the platinum film are both of nanometers. And it has been found from the AFM analysis that the surface roughness of the aluminum oxide film is large, the contact between alumina and platinum can be simplified to the hemispherical contact, just show in Fig. 9. Since platinum contains a large amount of free electrons, alumina is used as an insulator material, it contains few free electrons, so the interaction between electrons and phonons at the boundary is required. In the next part of this manuscript we will analyze this model by three methods to show that the heat generated by catalytic reaction is difficult to conduct through heat conduction, which also explains the cause of the large temperature gradient of the Nanofire.

In traditional heat transfer, energy passes through the solid interface in three ways: thermal conduction, thermal convection, and thermal radiation. Since the solid interface contact is mainly point contact, a double sphere model [41] can be used for simple simulation. It can be considered as the contact between a point of the upper sphere and the lower smooth sphere. The corresponding calculations are placed in Additional file 1. The double sphere model is based on Fourier’s law. But at the atomic or molecular scale, the boundary scattering effect of phonons is increased, the calculated thermal resistance (thermal conductivity) deviates greatly from the budget value of Fourier’s law, so the interface heat transfer must be reconsidered. Theories for studying microscopic interface heat transfer are mainly Acoustic Mismatch Model (AMM) [42] and Diffuse Mismatch Model (DMM) [43], we will detail these two methods in Additional file 1.

**Fig. 10:**
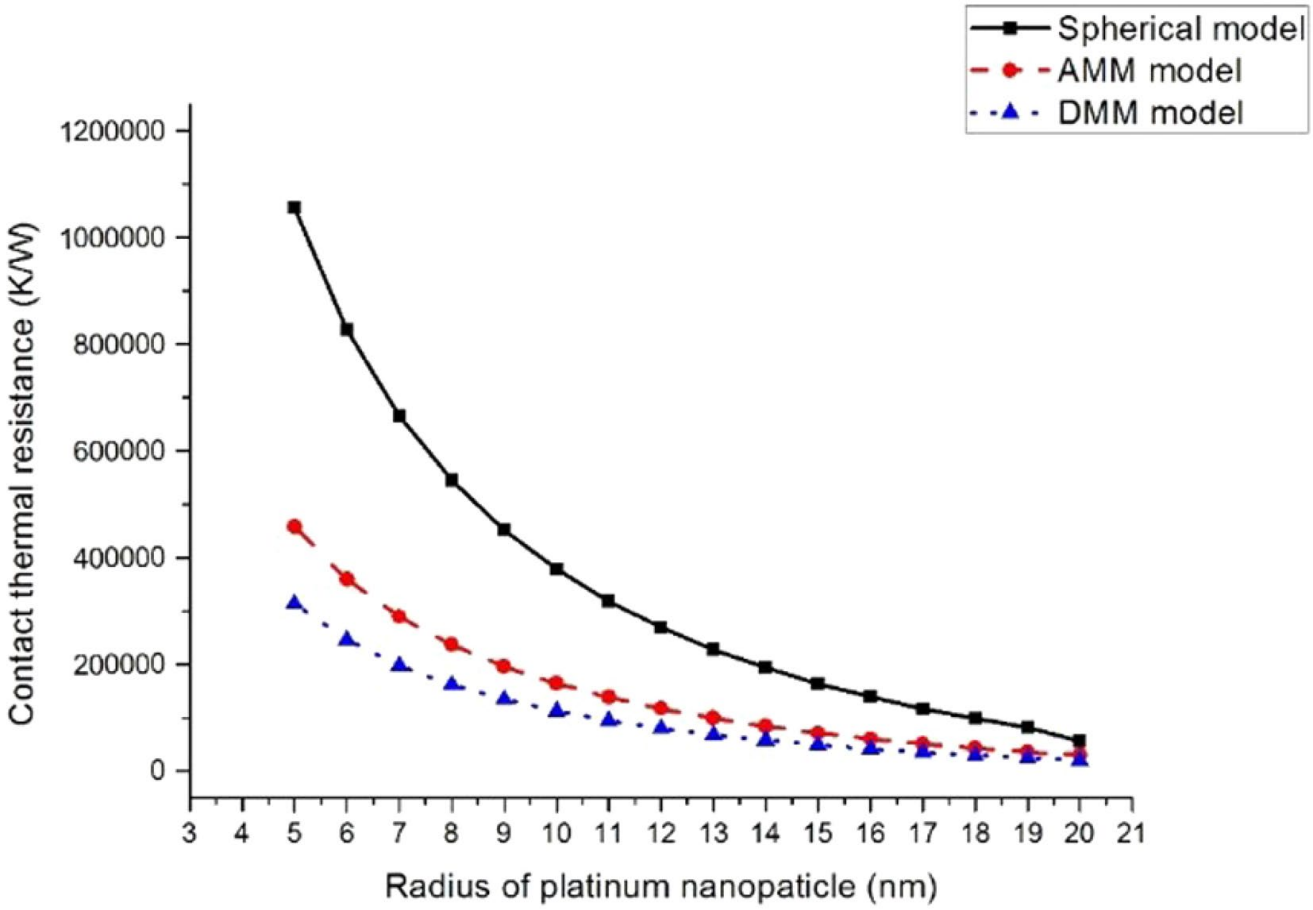
The contact thermal resistance calculated by three simulations

In this manuscript, we assume that the diameter of the platinum particles is 5–20 nm and the diameter of the alumina particles is 30 nm. The double sphere model, the AMM model and DMM model are used respectively to calculate the contact thermal resistance. The results are shown in Fig. 10.

It can be seen from the above simulation that when the diameter of the platinum particles gradually increases from 5 to 20 nm, the contact thermal resistance decreases whatever got by the double-sphere model, the AMM model and the DMM model. When the equivalent diameter of the platinum particles is about 5 nm, the magnitude of the contact thermal resistance is about 10^5^–10^6^ K/W. We can say that the amount of heat transferred by conduction is small. This also shows that the heat generated by catalytic combustion is rarely propagated downwards, most of which will accumulate on the surface of the catalyst, and a large thermal gradient is generated. At the same time, the above calculation results show that such a large thermal gradient is difficult to realize at the macro scale.

For thermoelectric materials, in order to improve its thermoelectric performance, that is, the ZT value, it is necessary to reduce its thermal conductivity while increasing its electrical conductivity. The ZT value of the thermoelectric material is calculated as follows:

$${\text{ZT}} = \frac{{\alpha^{2} T\sigma }}{\kappa }$$

where α is the Seebeck coefficient, σ is the conductivity, κ is the thermal conductivity, and T is the temperature. The above calculations also provide ideas for increasing the ZT value. Many groups now try to improve the thermoelectric properties of materials by designing multilayer low-dimensional thermoelectric materials. Multi-layer low-dimensional thermoelectric materials have more contact surfaces, so there will be a larger contact thermal resistance. Due to the difference in electron and phonon wavelengths, the presence of the interface has a greater influence on the thermal conductivity. It is a common method to improve the ZT value by increasing the interface of the material.

## Scale effects of heat and heat transfer

Since the 1980s, high-integration, high-density electronic devices have developed rapidly. At the same time, it has been found that there is no way for the traditional heat conduction law to solve the cooling problem of such devices. And at the nanometer scale, the feature size of the material may be smaller than the mean free path of the carrier, which causes the failure of the continuous medium hypothesis [44, 45]. So the macroscopic concepts and laws based on the continuous medium are no longer applicable, such as Fourier heat conduction law, N–S equation and so on.

**Fig. 11:**
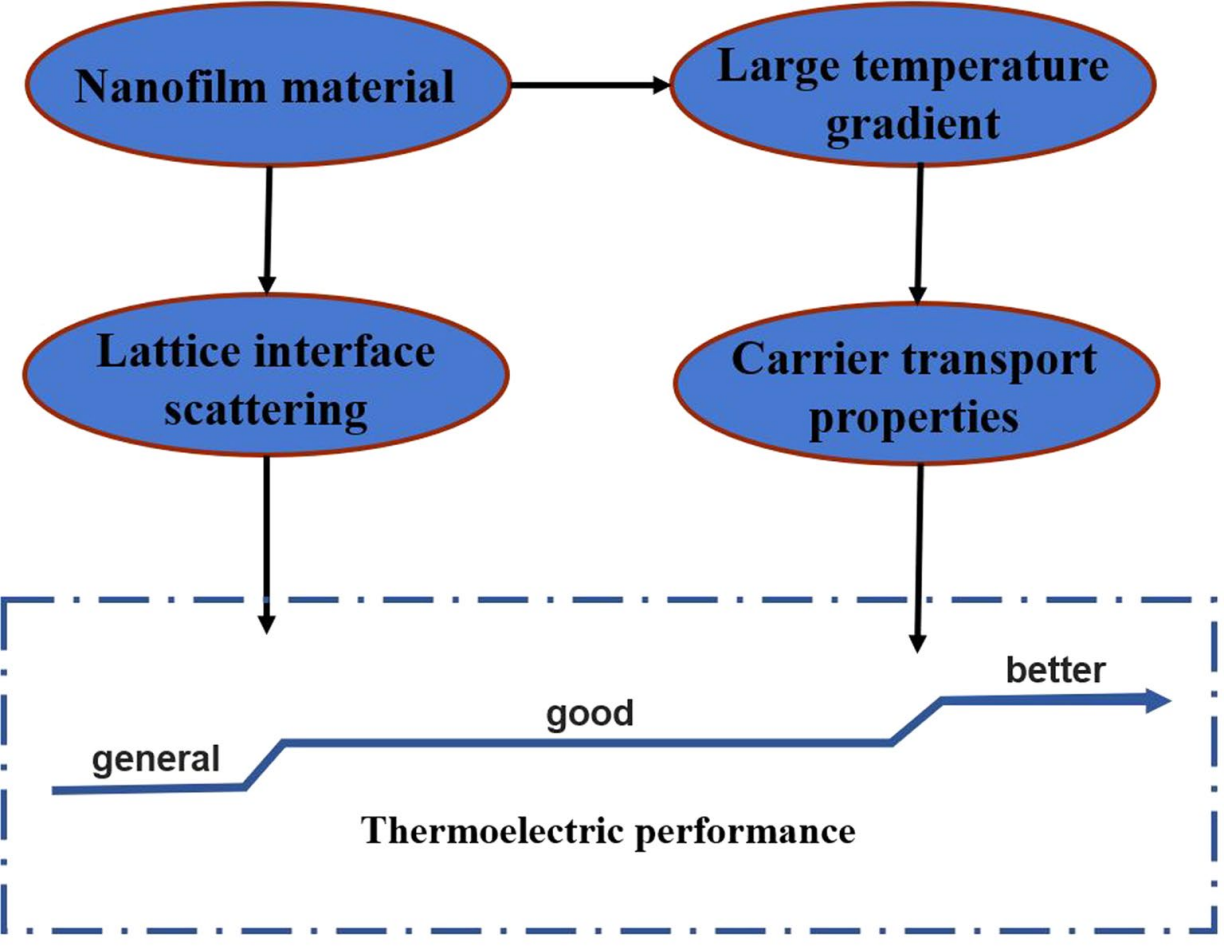
Scale effects of heat

The physical mechanism of the scale effect of thermal conductivity comes from two aspects: one is related to the length of the feature in the heat conduction problem [46]. On the other hand, the thermal conductivity is related to the grain size in the material [47]. Due to the increase in grain boundaries, the transport capacity is weakened and the thermal conductivity is reduced [48]. As shown in Fig. 11, due to the property of micro/nano scale heat conduction, extreme temperature gradients can be formed in nanofilm materials. And large temperature gradients affect the transport properties of carriers. At the same time, there are scattering effects at the material interface in the micro/nano multilayer film material. These together improve the thermoelectric properties of thermoelectric materials. According to the phenomena obtained in the research of Nanofire, we propose three scale effects of heat.

**Fig. 12:**
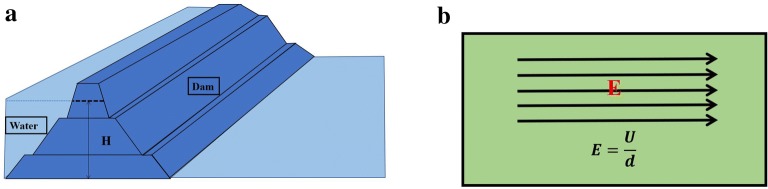
**a** The gravitational potential energy of water; **b** the electric field and electric field strength

A large thermal gradient can form a strong thermoelectric potential in a semiconductor material. The carrier is uniformly distributed in the conductor when the isolated conductor has not yet established a temperature difference. Once the temperature gradient is established, the carriers at the hot end have greater kinetic energy, begin to diffuse toward the cold end, and accumulate at the cold end. While a large temperature gradient can form a larger thermoelectric potential, we can compare the gravitational potential energy of water with the potential energy, as shown in Fig. 12. Figure 12a is a schematic diagram of the dam. As the water level increases, the gravitational potential of the water also gradually increases. So that the kinetic energy of the water will be large when the brake is opened, then there will be more electric energy. Figure 12b is the relationship between the electric field strength and the potential difference. When the potential difference is the same, the smaller the size is, the larger the electric field strength is. For Nanofire, the temperature gradient is the key to the transfer of chemical energy to electrical energy. In order to improve the conversion efficiency, the temperature difference is needed to be increased, while the size of the material is needed to be reduced. We can set the conversion efficiency to η ∝ ∇*Q*/*d*. ∇*Q* is the temperature different, and d is the thickness of thermoelectric materials.

Let’s talk about the influence of thermal gradient on carrier transport [49–51]. The transport properties of carriers such as phonons and electrons have a great influence on the properties of thermoelectric materials. For thermoelectric materials, lowering the thermal conductivity is to reduce the propagation speed of the heat carrier in the material. Different materials correspond to different propagation mechanisms, but they have some in common, such as the influence of temperature on their propagation. We can analyse this question from the perspective of conductors, insulators and semiconductors. The thermal conductivity is related to the motion state of the hot carrier in the material: the faster the thermal carrier moves, the greater the thermal conductivity is. At the same time, the collision or scattering effect of the hot carrier increases, and the thermal resistance increases accordingly. If the material is regarded as a road, then the hot carrier is the car driving on this road. At high temperatures, the carrier is subjected to a strong scattering effect, and the movement in the material is like a car driving at a high speed on a muddy road. On the contrary, at low temperatures, electron motion or lattice vibration will appear in a state similar to “freezing”, in which the movement of the hot carrier in the material is like a car traveling slowly on the highway. In summary, large temperature gradients cause carrier motion increase, consequently increase in scattering and collision, and vice versa. As mentioned above, the thermal resistance of the film material is very large. Therefore, a large temperature gradient has a greater influence on lattice vibration, and the performance of the thermoelectric material can be better improved.

**Fig. 13:**
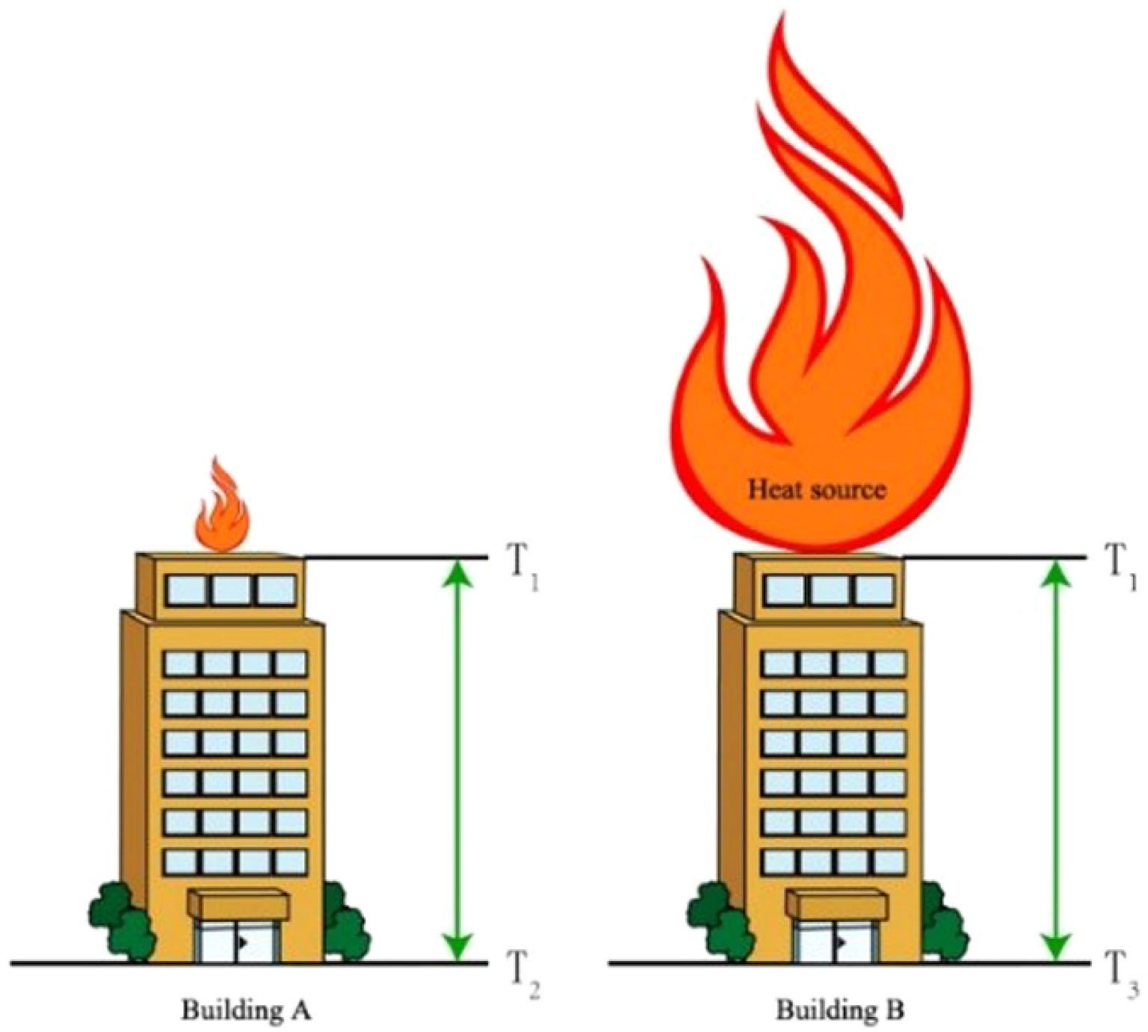
Effect of thermal size on thermal gradient build-up

Next, we explore the influence of the size of heat source. In the fourth part, we obtained the relationship between the thermal conductivity and the size of materials through simulation. It can be found that the thermal resistance is extremely high and the heat conduction can be neglected. It is found in the experiment the temperature of the substrate also rises slightly. We also simulated the thermal conductivity of the near-field radiation. Based on this, we can make a macro-scale analogy. As shown in Fig. 13, on top of two identical 5-story buildings. A heat source with a diameter of 10 cm, a height of 5 cm, a temperature of 1500 K, and the other heat source with a diameter of 5 m, a height of 15 m, and a temperature of 1500 K were placed. Then the temperature T1 at the top of the two buildings is the same, but due to the difference in the size of the heat source, there will be a big difference in the heat conduction and heat radiation. There will not be a significant increase of the floor temperature T2 of the building A, because it is little affected by the heat source. But the floor temperature T3 of the B building will increase significantly. As a result, a large temperature difference can be established between the upper and lower of the A building, and the thermal gradient of the B building cannot be established. This example shows that the establishment of the temperature difference is related to the relative size of the heat source. When the size of the heat source is small, the radiation effect to the surrounding space and the downward heat conduction effect will be greatly weakened. On the contrary, if the heat source of the roof is large in size, its radiation and heat conduction are strong, and the temperature in the surrounding space will also be strong, the temperature will rise soon.

The application of Nanofire is based on a large thermal gradient. In order to establish a large thermal gradient, the thickness of the catalytic layer (heat source) must be minimized. With the 20 nm ultra-thin film, graphical fire has a large thermal gradient, up to 6 × 10^8^ K/m. The key to achieving such a large thermal gradient is the size control of the heat source and the large longitudinal thermal resistance. The thickness of the platinum film catalyst is only 5 nm, which makes the heat radiation of the heat source downward is very low. According to the simulation calculation, the thermal resistance of the alumina and platinum films gradually decreases with the decrease of the size due to the contact thermal resistance increasing. For a 5 nm thin film catalyst, the thermal resistance is as high as 10^6^ K/W and the heat conduction is small. The weaker heat radiation effect and the larger contact thermal resistance together cause the combustion temperature of the upper surface of the catalyst to not propagate downward, and there is almost no temperature change on the lower surface of the catalyst, so that a large temperature gradient can be established. With the deepening of the research on thermal gradients, the problem of thermal scale will also enter everyone’s field of vision and become an indispensable part of thermodynamic research.

## Conclusion

Fire has been the fundamental base of human civilization over one million years. Macroscale fire and high temperature burring made current industrial world with environmental and other issues. There are needs to overcome dependency on the current energy system, meanwhile new energy currently in use like solar, wind and hydro has their drawbacks too. Therefore, there is room for alternative pathways, and Nanofire could potentially become one. Nanofire is a new energy utilization method different from macro-scale combustion, which has many excellent properties. We can prepare high-integration thermoelectric chip power generation boxes by means of micro–nano processing to achieve large-scale production and use of Nanofire. By studying Nanofire, we can find the scale effects of heat. The relative size of the heat source affects the establishment of the thermal gradient. The gradient affects the transport properties of the carriers in the material. The transport properties of the carriers determine the performance of the thermoelectric material, it is also related to the size of the material. In summary, we can find that nano-scale combustion can generate large thermal gradients, which will accelerate the movement of carriers such as phonons and electrons. There is strong phonon scattering in thin-film thermoelectric materials, so large thermal gradients combination of thin film thermoelectric materials enables efficient conversion of thermal energy to electrical energy. In this manuscript, we innovatively propose Nanofire and the scale effects of heat, but a lot of work remains to verify and supplement in the future. We believe that Nanofire will have a big effect.

Currently, our sub-micrometer thick thermoelectric array can produce continues electrical power with ultralow temperature difference (0.0001 K) that could be used to harvest almost all kinds of low-grade heat energy from surrounding. The large-scale effects of successfully deploying thermoelectric chips for power generation are difficult to oversee, as the potential to retrieve energy anytime, anywhere on a local level challenges the fundamental assumptions behind our energy grids and current distribution models. There is a potential for disruptive innovation by enabling large-scale off-grid solutions if Nanofire can be successfully matured. To assess such scenarios, future work on mapping stakeholders, their goals, actions and intentions, and the potential deployment pathways needs to be done. It is important to augment a business model approach, such as is common to any new technology, with simulated exercises to assess the consequences of this technology on the current societal infrastructures. Methods like gaming and participatory simulation in engineering systems [52] are proven to be effective mediators between important stakeholders to responsibly develop the technology into maturity.

## Additional file

**Additional file 1.** The simulation calculation of contact thermal resistance.
