# Supplementary material for: Nanofire and scale effects of heat
Source: Nano Converg. 2019 Feb 15;6:5. doi: 10.1186/s40580-019-0175-4 (PMC6376045; doi:10.1186/s40580-019-0175-4)
Supplement: Supplementary file 1 — Additional file 1. The simulation calculation of contact thermal resistance. [file 40580_2019_175_MOESM1_ESM.docx]

**Additional file 1**

**1 Hemispherical contact model**

Copper et al. proposed a classic hemispherical contact model

$$R_{i}=\frac{\varphi}{2a\lambda_{g}}$$

where$R_{i}$ is the single point contact thermal resistance；$a$ the contact radius of the contact point；$\lambda_{g}$ is the thermal conductivity；$\varphi$ the contact factor of the contact point, the expression is as follows:

$$\varphi=\left( 1-\frac{a}{b} \right)^{1.5}$$

Where b is the radius of curvature of the lower half of the material. Thus, the contact thermal resistance of this contact surface can be expressed as

$$R=\sum_{i=0}^{N} R_{i}$$

In order to further improve the model of the heat flow at the point contact, Mikic and Rohesnow also simplified the heat flow channel into a cylindrical channel. Gibson and Yovannovich further discussed and summary the size and shape features.

**2 AMM and DMM model**

The AMM model was first proposed by Khalatnikov and Mazo and Onsager in 1952 and 1955 respectively [5]. The model considers the interface as an ideal smooth plane, and assumes that the phonon is a plane wave in a continuum, and the phonon emits a specular surface at the interface. .
For two contacted objects, the net heat flux through the interface and the interface thermal resistance are:

$$q_{net}=q_{1\to2}-q_{2\to1}$$

$$R_{int}=\frac{T_{1}-T_{2}}{q_{net}}=\frac{\Delta T}{q_{net}}$$

Then the energy transmitted by the object 1 to the object 2 through the interface per unit time and unit area is

$$q_{1\to2}\left( T_{1} \right)=\frac{1}{2}\sum_{j} \int_{\theta=0}^{\frac{\pi}{2}} \int_{\omega=0}^{\omega_{d}} N_{1}\left( \omega,T_{1},j \right)\hbar\omega c_{1,j}\alpha_{1\to2}\left( \theta,j,\omega\right)\sin\left( \theta\right)\cos\left( \theta\right)d\omega d\theta$$

Where $j$ is the phonon mode; $N_{1}$the phonon state density of object 1; $\omega$the phonon frequency; $\theta$the phonon incident angle; $\hbar$the Planck number of the reduction; $\omega_{d}$the maximum phonon frequency; $c_{1,j}$ the phonon mode for the object 1 The phonon velocity at the time; $\alpha_{1\to2}\left( \theta,j,\omega\right)$ is the projection coefficient.

According to Debye theory

$$\hbar\omega_{d}=k_{b}\theta_{d}$$

The expression of phonon density is

$$N\left( \omega,T,j \right)=℘\left( \omega,j \right)\cdot f\left( \omega,T \right)$$

Where,

$$f\left( \omega,T \right)=\frac{1}{\left[ \exp\left( \frac{\hbar\omega}{k_{b}T} \right)-1 \right]}$$

Is the Bose-Einstein distribution coefficient; phonon density

$$℘\left( \omega,j \right)=\frac{\omega^{2}}{\left( 2\pi^{2}{c_{j}}^{3} \right)}$$

According to Debye theory, the phonon density can be written as

$N_{i}\left( \omega,T_{i},j \right)=\frac{\omega^{2}}{\left\{ 2\pi^{2}{c_{i,j}}^{3}\left[ \exp\left( \frac{\hbar\omega}{k_{b}T_{i}} \right)-1 \right] \right\}},i=1,2$

Wherein, $i=1,2$ representing object 1 and object 2 respectively; according to the phonon mismatch model assumption, the object transmission coefficients$\alpha_{1\to2}\left( \theta,j,\omega\right)$ and $\alpha_{2\to1}\left( \theta,j,\omega\right)$are both independent of the incident wavelength, and

$\alpha_{1\to2}\left( \theta,j,\omega\right)=\alpha_{2\to1}\left( \theta,j,\omega\right)$

According to the energy transfer coefficient independent of the angle of incidence, the transmission coefficient is defined here as

$\Gamma_{1\to2}=\int_{0}^{\theta_{1c}} \alpha_{1\to2}\left( \theta,j \right)\sin\left( \theta\right)\cos\left( \theta\right)d\theta$

From this, we can get the expression of heat flux density and the expression of interface thermal resistance

$q_{net,AMM}=\frac{\pi^{2}}{15}\frac{{k_{b}}^{4}}{\hbar^{3}}\frac{\Gamma_{1\to2}}{{c_{1l}}^{2}}T_{2}^{3}\Delta T$

$$R_{int, AMM}=\left[ \frac{\pi^{2}}{15}\frac{{k_{b}}^{4}}{\hbar^{3}}\frac{\Gamma_{1\to2}}{{c_{1l}}^{2}} \right]T_{2}^{-3}$$

In the high temperature region, the phonon wavelength decreases with increasing temperature. When the wavelength of the dominant phonon in the crystal is reduced to be equivalent to the interface roughness, the ideal interface assumption of AMM is no longer valid. The effect of interface roughness on phonon interface scattering must be considered, that is, the scattering mismatch model. That is to say, the phonon scatters at the interface rather than the reflection, and the probability that a phonon enters material 1 or material 2 is proportional to the density of states corresponding to the phonon in the material, and is subject to the principle of fine balance. It has nothing to do with where the phonon comes from, that is, regardless of the angle of incidence, thus:

$\alpha_{1\to2}\left( \theta,j,\omega\right)+\alpha_{2\to1}\left( \theta,j,\omega\right)=1$

This gives the net heat flux density of the interface.

$q_{net,DMM}=\frac{k_{b}^{4}}{8\pi^{2}\hbar^{3}}\left\{ T_{1}^{4}\sum_{j} c_{1,j}^{-2}\int_{0}^{\frac{\hbar\omega}{k_{b}T_{1}}} \frac{z^{3}\alpha_{1\to2}dz}{\exp\left( z \right)-1}-T_{2}^{4}\sum_{j} c_{2,j}^{-2}\int_{0}^{\frac{\hbar\omega}{k_{b}T_{2}}} \frac{z^{3}\alpha_{2\to1}dz}{\exp\left( z \right)-1} \right\}$

Where $z=\frac{\hbar\omega}{k_{b}T}$，$\omega_{d}=\min\left( \omega_{d1},\omega_{d2} \right)$，

$\omega_{d1}$、$\omega_{d2}$ are the Debye frequencies of object 1 and object 2, respectively. Can be further obtained

$\alpha_{1\to2}\left( \omega\right)=\frac{\sum_{j} N_{2}\left( \omega,T,j \right)c_{2,j}}{\left[ \sum_{j} N_{1}\left( \omega,T,j \right)c_{1,j}+\sum_{j} N_{2}\left( \omega,T,j \right)c_{2,j} \right]}$

Substituting phonon density, there is

$\alpha_{1\to2}\left( \omega\right)=\frac{\sum_{j} c_{2,j}^{-2}}{\left[ \sum_{j} c_{1,j}^{-2}+\sum_{j} c_{2,j}^{-2} \right]}$

Correspondingly, the heat flux density through the interface

$q_{net,DMM}=\frac{\pi^{2}}{120}\frac{k_{b}^{4}}{\hbar^{3}}\left( T_{1}^{4}c_{1}^{-2}\alpha_{1\to2}-T_{2}^{4}c_{2}^{-2}\alpha_{2\to1} \right)$

Interface thermal resistance

$R_{int}=\left[ \frac{\pi^{2}}{30}\frac{k_{b}^{4}}{\hbar^{3}}c_{1}^{-2}\alpha_{1\to2} \right]^{-1}T_{2}^{-3}$
